# Forced Exercise Enhances Functional Recovery after Focal Cerebral Ischemia in Spontaneously Hypertensive Rats

**DOI:** 10.3390/brainsci2040483

**Published:** 2012-10-16

**Authors:** Sookyoung Park, Jinhee Shin, Yunkyung Hong, Sunmi Kim, Seunghoon Lee, Kanghui Park, Tserentogtokh Lkhagvasuren, Sang-Rae Lee, Kyu-Tae Chang, Yonggeun Hong

**Affiliations:** 1 Cardiovascular & Metabolic Disease Center, College of Biomedical Science & Engineering, Inje University, Gimhae 621-749, Korea; Email: charm-soo@hanmail.net (S.P.); jani222@hanmail.net (J.S.); bluesky1346@hanmail.net (S.K.); tserentog_l@yahoo.com (T.L.); 2 Department of Rehabilitation Science in Interdisciplinary PhD Program, Graduate School of Inje University, Gimhae 621-749, Korea; Email: dangmoo777@naver.com (Y.H.); stormyboy@nate.com (S.L.); jspt95@hanmail.net (K.P.); 3 National Primate Research Center (NPRC), Korea Research Institute of Bioscience and Biotechnology (KRIBB), Ochang 363-883, Korea; Email: srlee@kribb.re.kr

**Keywords:** focal cerebral ischemia, middle cerebral artery occlusion, caveolins, autophagy, forced exercise, spontaneously hypertensive rat

## Abstract

Caveolin is the principal protein of caveolae and has been implicated in the pathogenesis of cerebral ischemia. To investigate whether changed expression of caveolins has a pivotal role in focal cerebral ischemia, we induced middle cerebral artery occlusion (MCAo)-reperfusion and examined expression of caveolins, inflammatory activation markers, and mediators of autophagic cell death. We also treated MCAo rats with forced exercise to determine its effects on neurological outcome. Particularly, spontaneously hypertensive rats (SHR) and normotensive Wistar-Kyoto (WKY) rats were used to compare the effects of hypertension on focal cerebral ischemia. All MCAo groups showed neurological deficiencies, motor dysfunction, and disruption of balancing ability; however, these pathological changes were more severe in SHR than WKY rats. Expression of caveolins was decreased in MCAo brain tissue, whereas the levels of iNOS and glial fibrillary acidic protein (GFAP) increased. Additionally, LC3-II and beclin-1 levels were elevated in the MCAo groups. Forced exercise attenuated both molecular and behavioral changes in MCAo animals, but SHR rats showed delayed functional recovery and residual molecular changes when compared to WKY rats. These results suggest that forced exercise may be beneficial for promoting functional recovery following cerebral ischemia through caveolin-dependent mechanisms or interactions between caveolins and these signaling molecules in ischemic brain regions.

## 1. Introduction

Cerebrovascular disease (CVD) is the third leading cause of death and long-term disability worldwide [1]. It is also the most prevalent neurological disorder in terms of both morbidity and mortality [2]. CVD may have extremely detrimental consequences as 80% of patients with this disease may develop cerebral ischemia [3]. Cerebral ischemia results from the loss of blood supply to part of the brain caused by various mechanisms such as atherothrombosis, cardioembolism, or hemodynamic compromise; and loss of brain function develops rapidly afterwards [1,2,4]. Risk factors for cerebral ischemia include advanced age, hypertension, previous stroke or transient ischemic attack (TIA), diabetes, high cholesterol, and atrial fibrillation [1,2,3,4]. Hypertension is the most important risk factor for exacerbations of cerebral ischemia and is associated with endothelial dysfunction and increased cardiovascular risk [2,4,5]. Therefore, blood pressure is thought to be a significant determinant of stroke risk in both normotensive and hypertensive populations [6].

Multiple mechanisms are involved in the development of hypertension and cerebrovascular disease. Recent studies reported that caveolae and caveolins play critical roles in cardiovascular pathophysiology [7,8,9,10]. The caveolae are omega-shaped membrane invaginations present essentially in all mammalian cell types. Moreover, caveolae are formed in a caveolin-dependent manner [11]. Caveolins are 21–24-kDa integral, cholesterol-binding membrane proteins that interact with a number of caveolae-associated signaling molecules [11,12,13,14]. Caveolae and caveolins are particularly important for blood pressure regulation by the endothelium [8]. Additionally, caveolin-1 deficiency leads to hyperactive endothelial nitric oxide synthase (eNOS) and results in experimental forms of pulmonary hypertension (PH) [15]. The downregulation of caveolins induces a number of clinical symptoms. In particular, caveolins appear to act in the pathogenesis of cerebral ischemia. Remodeling and plasticity from cerebral ischemia require cholesterol redistribution and synthesis for the formation of new neuronal cell membrane component therefore cholesterol plays a central role in membrane compartmentalization [14,16]. Cholesterol is localized in the caveolae of cellular membranes, and caveolins bind cholesterol and transport it to injured areas [17]. These properties of caveolins suggest that they play a critical role in cell membrane remodeling and synaptic plasticity from cerebral ischemia [18].

To investigate the pathology of cerebral ischemia, various animal studies have been conducted. Longa *et al*. reported that an animal model of middle cerebral artery occlusion-reperfusion (MCAo) microsurgery produced focal cerebral ischemia and neurological deficiencies [19,20] and has been used widely to investigate the pathogenesis of cerebral ischemia [5,21,22]. In the MCAo rat, nitric oxide (NO) modulates the expression of caveolin-1 in the brain, and the loss of caveolin-1 is associated with NO production in the ischemic brain [23]. NO is a signaling molecule that binds caveolins in caveolae and is a diffusible cellular mediator generated by nitric oxide synthase (NOS) [23,24]. Three NOS isoforms exist: neuronal NOS (nNOS), inducible NOS (iNOS), and endothelial NOS (eNOS) [24,25]. At physiological concentrations (levels less than 10 nmol/L), NO produced by eNOS is essential for neuronal communication and regulation of vascular tone [23,26]. However, during cerebral ischemia, excess NO created by the activation of iNOS by macrophages and the calcium-dependent activation of nNOS may contribute to pathology in the ischemic brain [24,25,26]. In addition, high NO concentrations can cause inflammation and neuronal apoptotic cell death, leading to increased infarction size [26,27].

The contributions of specific cell death pathways, including apoptosis and necrosis, have been extensively studied in cerebral ischemia. Recent reports also describe autophagic cell death meeting the criteria for type 2 programmed cell-death morphology following hypoxia-ischemic damage [28,29,30]. Induction of autophagy after focal cerebral ischemia has been associated with the upregulation of Beclin-1 and autophagy-like cell death [30]. Autophagic death is distinct from apoptotic (type 1) death; it is an intracellular bulk degradation process in which cytosolic, long-lived proteins and organelles are degraded and recycled [31]. The autolysosomal degradation of membrane lipids and proteins generates free fatty acids and amino acids, which can be reused to maintain mitochondrial ATP production, protein synthesis and promote cell survival [32]. Autophagy may also protect neuronal cells by eliminating damaged structures and delivering them to the lysosome for degradation [29]. Therefore, it is not clear whether autophagy is beneficial or damaging in the context of cerebral ischemia. Changes in caveolin expression has also been associated with autophagy; there was increased activation of autophagy in the cardiac tissue of caveolin-1-deficient mice [33]. Additionally, Class III PI3K-related autophagosome formation involves a binding protein in mammalian cell membrane caveolae [34]. It is not known whether autophagy involves cross-talk with caveolins during cerebral ischemia. Furthermore, the nature of the interaction between caveolins and NOS signaling after cerebral ischemia is not well understood. 

To identify the effect of hypertension on focal cerebral ischemia, we subjected normotensive Wistar Kyoto (WKY) rats and spontaneously hypertensive rats (SHR) to MCAo microsurgery. Forced exercise on a treadmill has been used widely to investigate the repair of ischemic brain damage. Therefore, the WKY- and SHR-MCAo rats were subjected to forced exercise to test whether this treatment attenuates typical neurological deficiency and has neuroprotective effects on cerebral ischemia. In this study, we specifically focused on the role of caveolins and NOS as key regulators and examined the effects of alterations in autophagy on the pathophysiology of cerebral ischemia.

## 2. Results

### 2.1. Sequential Tracing of Physiological Characteristics of WKY and SHR Rats

**Figure 1:**
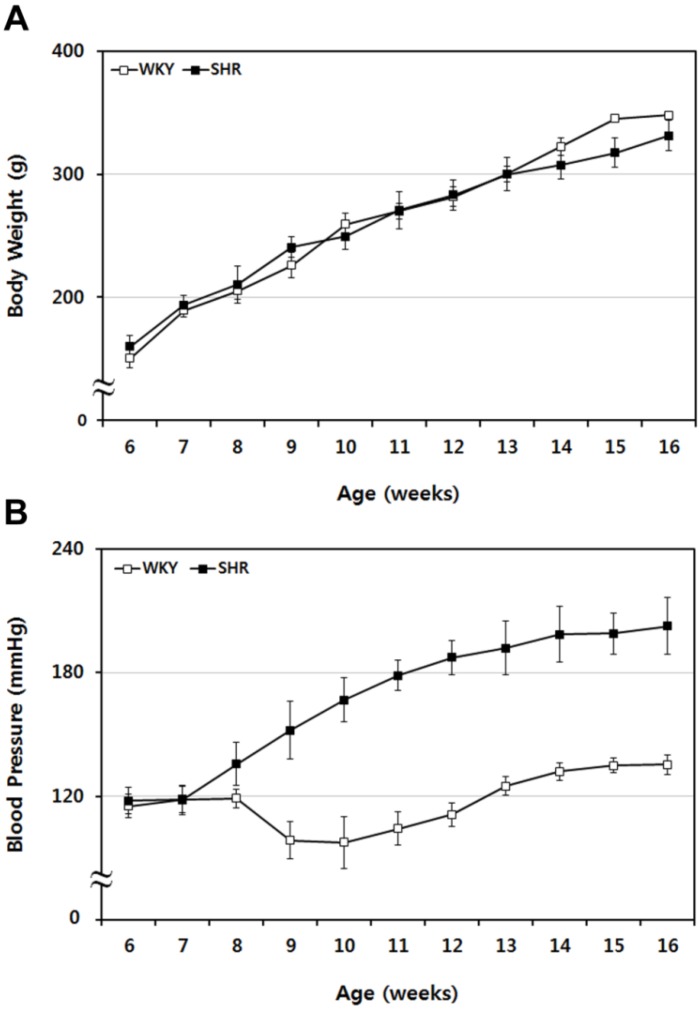
Sequential tracing of body weight and blood pressure in WKY and SHR rats. (**A**) Body weight of WKY and SHR rats was measured for 10 weeks and compared. The two groups showed similar patterns of weight gain. (**B**) Blood pressure in WKY and SHR rats was also assessed periodically over 10 weeks. The systolic blood pressure of SHR rats increased markedly, compared with the normotensive WKY rats. Data shown are means ± SEM.

WKY and SHR rat body weight and blood pressure were traced periodically from 6 to 16 weeks of age. The body weight and blood pressure of WKY and SHR rats were measured and compared weekly. Body weight gain followed a similar pattern between groups (see Figure 1A); conversely, the blood pressure pattern was different between groups. Systolic blood pressure of SHR rats was markedly increased compared to normotensive WKY rats (see Figure 1B). We demonstrated that hypertension (≥180 mmHg) was evident in SHR rats from 12 weeks of age. Based on these findings, SHR rats with approximately 200 mmHg systolic blood pressure and age-matched WKY rats (12 weeks of age) were used as controls for MCAo surgery. 

### 2.2. 2,3,5-Triphenyltetrazolium Chloride (TTC)-Defined Ischemic Lesion Volume in WKY and SHR Rats after MCAo Surgery

**Figure 2:**
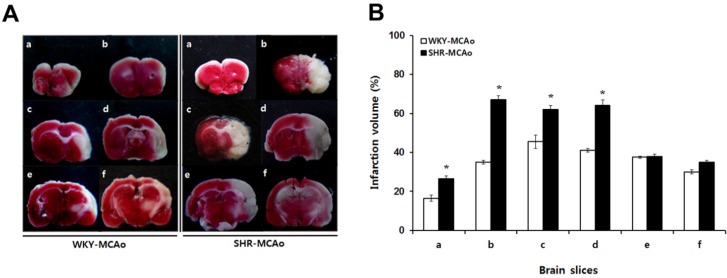
2,3,5-triphenyltetrazolium chloride (TTC)-defined volume of ischemic lesions in WKY and SHR rats after middle cerebral artery occlusion (MCAo) surgery. (**A**) Representative TTC staining of brain slices in rats after MCAo. After 24 h, WKY and SHR rats were sacrificed, and their brains were removed and cut into coronal slices. (**B**) Lesion volumes were determined for all brain slices. For each slice, the lesion volume is expressed as the percentage of the total ipsilateral hemispheric slice volume. Brains were cut into coronal slices of 2-mm thickness between the bregma levels of +4 mm (anterior) and −6 mm (posterior), yielding six coronal slices. Brain slices were labeled a to f in sequence. Data shown are means ± SEM, * *p* < 0.05 in WKY-MCAo and SHR-MCAo.

To determine the effects of hypertension on cerebral infarction due to MCAo, MCAo rats in WKY and SHR were sacrificed and compared cerebral infarction volume at 24 h after MCAo surgery. The brains were removed and cut into six 2-mm-thick coronal slices between the bregma levels of +4 mm (anterior) and −6 mm (posterior). Lesion volumes were determined for all brain slices and are expressed as a percent of the total ipsilateral hemispheric slice volume (see Figure 2A). The cerebral infarction volume of the first brain slice was 16.5% ± 1.50% and 26.5% ± 1.50% in WKY and SHR rats, respectively (*p* < 0.05). The cerebral infarction volume of the second brain slice was 35% ± 1.0% and 67% ± 2.0% in WKY and SHR rats (*p* < 0.05), respectively. Lesions were also evident in the third (45.5% ± 3.50% and 62% ± 2.0%) and fourth brain slices (41% ± 1.0% and 64% ± 3.0%) in both WKY and SHR rats (*p* < 0.05), respectively (see Figure 2B). These results demonstrated that SHR rats had more severe cerebral infarction volumes as compared with control WKY rats after MCAo surgery. Thus, hypertension appears to be a risk factor affecting extent of lesion size after focal cerebral ischemia. 

### 2.3. Neurological Deficiency and Motor Dysfunction Induced by Focal Cerebral Ischemia Were Reduced by Forced Exercise

**Figure 3:**
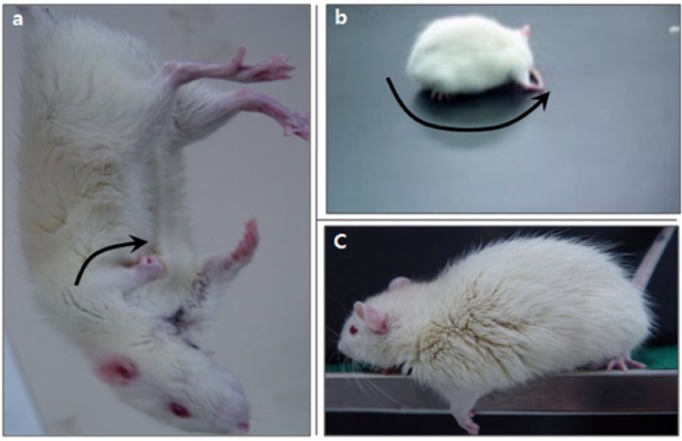
Neurological dysfunction in WKY and SHR rats with sham or MCAo surgery. (**a**) Paralytic forelimb was flexed during tail suspension (arrow). (**b**) Spontaneous circling was shown on the paretic side. (**c**) Decreased grip power of the left forelimb when the tail was pulled.

WKY and SHR rats with MCAo showed typical neurologic and motor dysfunction. The paretic forelimb was flexed during tail suspension (Figure 3a) and spontaneous circling (Figure 3b) was noted to the paretic side. Grip strength in the paretic forelimb (Figure 3c) was also decreased while the tail was pulled. Neurological deficiency was scored by mNSS, and these scores in MCAo WKY and SHR rats were increased compared with those of the Sham groups (*p* < 0.01). Also, the mNSS scores in the MCAo + Ex group were significantly lower compared to untreated WKY MCAo animals (*p* < 0.05), but not SHR MCAo rats. In addition, mNSS scores of MCAo group in SHR were higher than WKY-MCAo groups (*p* < 0.05), and also WKY MCAo + Ex group mNSS scores were improved compared with the SHR MCAo + Ex group (*p* < 0.05). This suggests that SHR showed more severe neurological disability due to MCAo surgery than WKY, also recovery from neurological deficiency in SHR rats was delayed compared to that in WKY rats (see Table 1). The beam-walking test scores of the MCAo groups were lower compared to WKY and SHR Sham groups (*p* < 0.01). Beam-walking test scores in the WKY MCAo + Ex group improved significantly compared to MCAo only WKY controls (*p* < 0.05). However, no improvement in beam-walking score was noted in SHR rats after forced exercise (see Table 1). The hindlimb stride width of MCAo groups increased compared to WKY and SHR Sham groups (*p* < 0.01). The hindlimb stride width of the MCAo + Ex groups decreased significantly compared to the WKY and SHR MCAo groups (*p* < 0.01) (see Table 1). These results suggest that forced exercise has therapeutic effects on neurological deficiency and motor dysfunction due to MCAo. 

**Table 1:** Mann-Whitney analysis of mNSS score and motor dysfunction in WKY and SHR rats with sham or MCAo surgery. mNSS score, balance beam scores and hindlimb stride width were assessed in WKY and SHR rats after forced treadmill exercise for 4 weeks.

	WKY				SHR			
	Sham	Sham + Ex	MCAo	MCAo + Ex	Sham	Sham + Ex	MCAo	MCAo + Ex
mNSS (Score)	1 (0–2)	1 (0–2)	11 (10–12) **	8 (8–10) ^#^	1 (1–2)	1 (1–2)	13 (12–14) **^,a^	12 (9–12) ^b^
Beam-walking test (Score)	6 (5–6)	6 (5–6)	3 (2–4) **	4 (3–5) ^#^	6 (5–6)	6 (5–6)	3 (2–4) **	4 (2–5)
Hindlimb stride width (cm)	5.2 (4.8–6.0)	5.2 (5.1–5.7)	7.1 (6.4–7.2) **	5.8 (5.6–6.1) ^##^	5.1 (4.9–5.4)	5.3 (4.7–5.8)	7.4 (7.0–8.1) **	6.1 (5.7–7.1) ^#^^#^

All data were represented by median value and range; ** *p* < 0.01 between Sham and MCAo (Mann-Whitney two tailed test); ^#^^#^* p* < 0.01 and ^#^* p* < 0.05 between MCAo and MCAo + Ex (Mann-Whitney two tailed test); ^a ^*p* < 0.05 between WKY-MCAo and SHR-MCAo (Mann-Whitney two tailed test); ^b ^*p* < 0.05 between WKY-MCAo + Ex and SHR-MCAo + Ex (Mann-Whitney two tailed test).

### 2.4. Altered Expression of Caveolin and NOS Isoforms Due to Focal Cerebral Ischemia in WKY and SHR Rats

**Figure 4:**
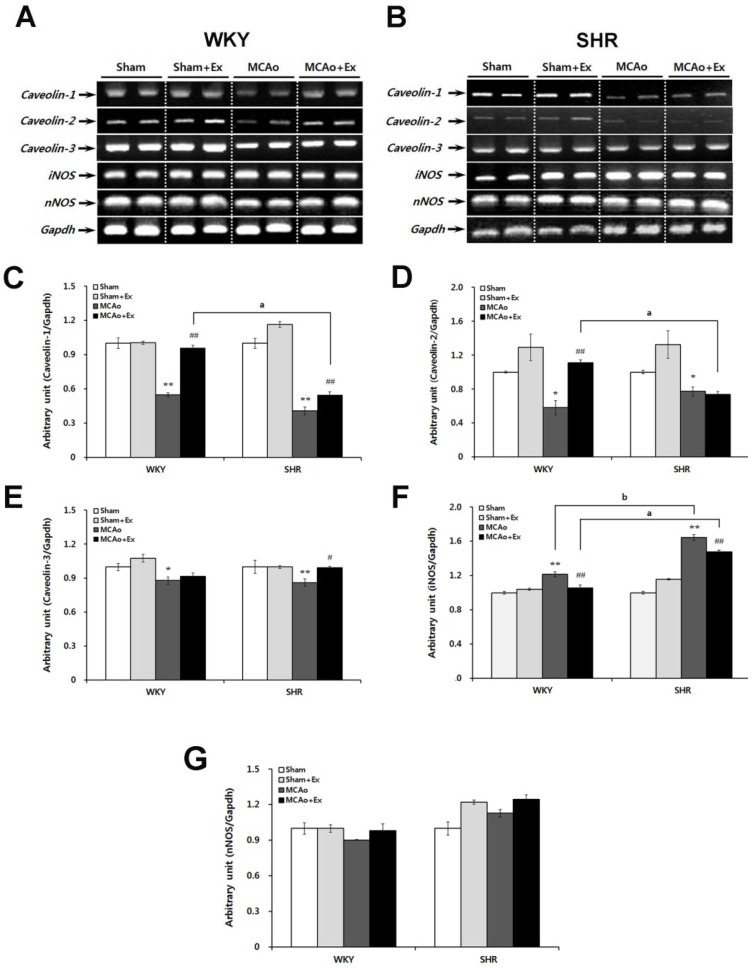
Altered expression of caveolins and NOS isoforms in WKY and SHR rats after MCAo surgery. (**A**) Electrophoresis of caveolin-1, -2, -3, nNOS, and iNOS in brain tissue of WKY rats and (**B**) SHR rats with MCAo surgery. (**C**) Expression of caveolin-1 mRNA in the MCAo group was significantly decreased compared with that in the sham group in WKY and SHR rats. Also, caveolin-1 mRNA was increased in SHR rats after the treadmill exercise. (**D**) Caveolin-2 mRNA levels were increased in the MCAo + Ex group in WKY rats after exercise. (**E**) Caveolin-3 mRNA was decreased in the MCAo group in WKY rats, and it increased after exercise. (**F**) Expression of iNOS mRNA was increased in the MCAo group of WKY and SHR rats. (**G**) Expression of nNOS mRNA in the MCAo groups of WKY and SHR rats was decreased. Data shown are means ± SEM. ** *p* < 0.01, compared with sham group; ^#^* p* < 0.05, ^## ^*p* < 0.01 compared with MCAo group; ^a ^*p* < 0.01 between MCAo + Ex WKY and SHR groups; ^b ^*p* < 0.01 between WKY and SHR MCAo groups.

RT-PCR analysis was performed to investigate alterations in caveolin and NOS isoforms and to evaluate the effect of forced exercise in WKY and SHR rats after MCAo surgery. Forced treadmill exercise (20–25 m/s, 30 min, twice daily) began 3 days post-injury and lasted for 4 weeks, after which the assessments were performed (*i.e.*, 4 weeks after injury). The caveolin-1, -2, and -3, iNOS, and nNOS mRNA levels were analyzed in core ischemic brain areas of WKY and SHR rats (see Figure 4A,B). The MCAo groups had dramatically down-regulated caveolin-1 mRNA levels in core areas compared to the WKY and SHR Sham groups (*p* < 0.01). After forced exercise, the caveolin-1 mRNA level increased significantly in WKY and SHR MCAo + Ex groups (*p* < 0.01). However, the caveolin-1 mRNA level in the SHR MCAo + Ex group was significantly lower than that in the WKY MCAo + Ex group (see Figure 4C; *p* < 0.05). The caveolin-2 mRNA level decreased in the MCAo groups compared with Sham groups in both WKY and SHR rats (*p* < 0.05). The caveolin-2 mRNA level was significantly increased only in the WKY MCAo + Ex group (*p* < 0.01), but not in SHR rats. Additionally, the caveolin-2 mRNA level in the SHR MCAo + Ex group was significantly lower than that in the WKY MCAo + Ex group (*p* < 0.05; see Figure 4D). MCAo surgery led to reduced caveolin-3 mRNA expression in both WKY (*p* < 0.05) and SHR rats (*p* < 0.01). Caveolin-3 was increased in the SHR MCAo + Ex group (*p* < 0.05). Caveolin-3 mRNA was predominantly expressed in the brain tissue of all groups compared with caveolin-1 and -2 (see Figure 4E). These results indicate that forced exercise has beneficial effects on caveolin expression in MCAo rats, whereas hypertension may have negative effects on recovery of caveolin expression in MCAo rats, particularly the caveolin-1 and -2 isoforms. NO produced by activated iNOS stimulates neuronal apoptotic cell death; therefore, we confirmed iNOS expression in WKY and SHR rats after MCAo surgery. Both WKY and SHR MCAo groups had significant increases in iNOS mRNA levels (*p* < 0.01). The iNOS mRNA level in the SHR MCAo group was up-regulated compared to the WKY MCAo group (*p* < 0.05). Forced exercise diminished the iNOS mRNA level in both WKY and SHR rats (*p* < 0.01); however, despite this change, iNOS mRNA level in the SHR MCAo + Ex group was higher than that in the WKY MCAo + Ex group (see Figure 4F; *p* < 0.05). These results suggest that hypertension may exacerbate iNOS expression in focal cerebral ischemia. nNOS mRNA levels in both MCAo groups were not significantly different compared with the Sham groups. The nNOS mRNA levels in both MCAo + Ex groups were slightly increased, but these changes were not significant (see Figure 4G).

### 2.5. Effects of Forced Exercise on Caveolin-1 and iNOS Interaction and Autophagy Signaling after Focal Cerebral Ischemic Injury

**Figure 5:**
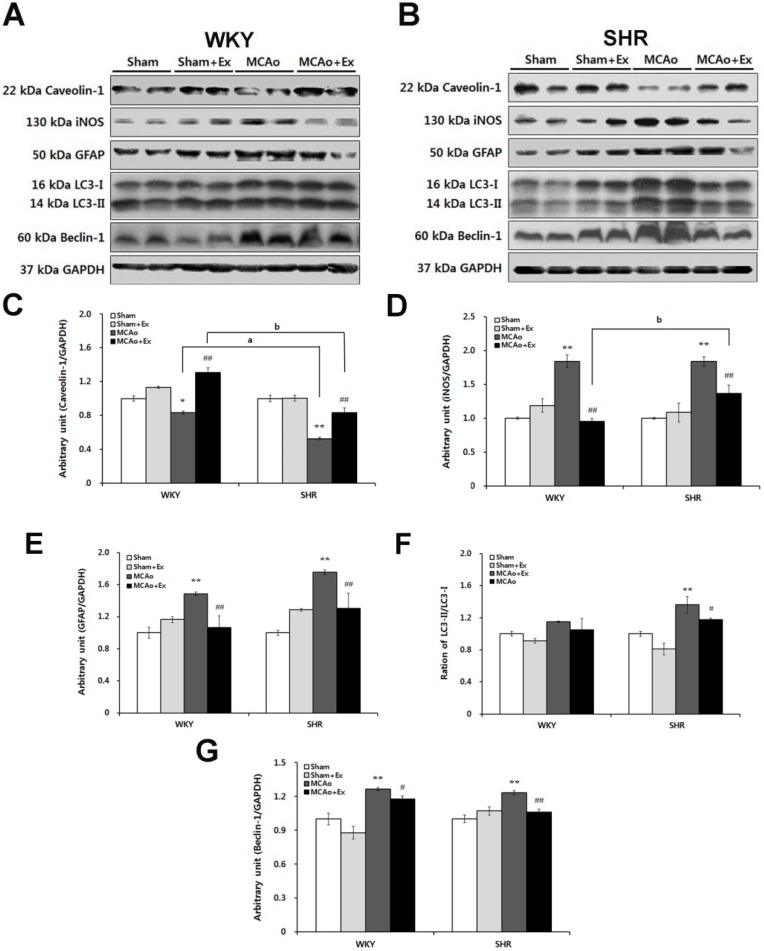
Effects of forced exercise on interaction of caveolin-1 and iNOS and autophagy signaling after focal cerebral ischemic injury. (**A**) Western blot analysis for caveolin-1, iNOS, glial fibrillary acidic protein (GFAP), and autophagy signaling in brain tissue of WKY rats and (**B**) SHR rats with MCAo surgery. (**C**) Caveolin-1 protein levels were down-regulated after MCAo, and they increased after exercise (*p* < 0.05). (**D**) iNOS protein levels were increased in WKY and SHR rats after focal cerebral ischemia. SHR rats showed a larger increase in iNOS protein levels compared with that in WKY rats. (**E**) GFAP protein levels were assessed in WKY and SHR rats after MCAo surgery. We found up-regulation in the MCAo group of both WKY and SHR rats, whereas we observed down-regulation in the MCAo + Ex groups. (**F**) The conversion ratio of LC3 I to LC3 II and (**G**) beclin-1 were increased by MCAo surgery and down-regulated after exercise (*p* < 0.05). Data shown are means ± SEM. * *p* < 0.05, ** *p* < 0.01 compared with sham group; ^# ^*p* < 0.05, ^## ^*p* < 0.01 compared with MCAo group; ^a ^*p* < 0.01 between WKY and SHR MCAo groups; ^b ^*p* < 0.01 between WKY and SHR MCAo + Ex groups.

Western blotting was used to determine the effects of forced exercise on protein expression related to caveolin-1, iNOS, autophagy, and GFAP signaling in brain tissue of WKY and SHR rats (Figure 5A,B). Caveolin-1 is strongly related to cerebral ischemic injury [35] and interacts with NO [23]. In this study, the caveolin-1 mRNA level was dramatically altered in both WKY and SHR MCAo groups. Therefore, we confirmed the expression of caveolin-1 and iNOS protein in brain tissue of WKY and SHR rats. 

The caveolin-1 protein level was decreased in the WKY (*p* < 0.05) and SHR (*p* < 0.01) MCAo groups. Specifically, SHR MCAo rats had significantly lower caveolin-1 protein expression than that in WKY rats (*p* < 0.01). After 4 weeks of forced exercise, the caveolin-1 protein level increased in both WKY and SHR rats (*p* < 0.01), but the SHR rat caveolin-1 protein level was significantly lower than that of WKY rats (*p* < 0.01; see Figure 5C). iNOS, a marker of secondary inflammation, was also confirmed. The iNOS protein level in the MCAo group was up-regulated compared with that in the WKY and SHR Sham groups (*p* < 0.01). Forced exercise dramatically attenuated iNOS protein expression in both the WKY and SHR MCAo + Ex groups (*p* < 0.01). However, the SHR MCAo + Ex group had increased iNOS protein levels compared with that in WKY rats after forced exercise (*p* < 0.01; see Figure 5D). In the WKY and SHR MCAo groups, glial fibrillary acidic protein (GFAP) levels increased (*p* < 0.01). Both the WKY and SHR MCAo + Ex groups had a decreased GFAP level after forced exercise (*p* < 0.01; see Figure 5E). These results indicate that focal cerebral ischemia induced caveolin-1 protein deficiency and increased expression of iNOS and GFAP proteins. Therefore, loss of caveolin-1 is associated with NO production and secondary inflammatory markers in the ischemic brain. Furthermore, SHR rats showed significant loss of caveolin-1 protein levels and induction of iNOS and GFAP levels compared with WKY rats, which suggests that hypertension aggravates inflammatory damage after cerebral ischemic injury. To evaluate autophagy activation induced by focal cerebral ischemia, LC3 and beclin-1 protein levels were confirmed in WKY and SHR rats. In injured SHR brain regions, the LC3-II protein level was markedly elevated compared with that in Sham groups (*p* < 0.01; see Figure 5F). Forced exercise markedly attenuated these effects, although in the SHR MCAo + Ex group, LC3-II protein showed slightly higher expression compared with that in the WKY MCAo + Ex group. Another marker of autophagic cell death, beclin-1, was also elevated over normal Sham levels in both WKY and SHR rats (*p* < 0.01). The beclin-1 protein levels were reduced after exercise in WKY (*p* < 0.05) and SHR rats (*p* < 0.01; see Figure 5G). These results indicate that focal cerebral ischemia stimulates autophagic cell death and that these effects are reduced by forced exercise.

## 3. Discussion

In the current study, we demonstrated altered expression of caveolins and regulatory signaling molecules due to focal cerebral ischemia and neuroprotective effects of forced exercise in MCAo rats. Additionally, to identify the influences of hypertension on ischemic brain damage, systolic blood pressures in WKY and SHR rats were followed from 6 to 16 weeks of age. We demonstrated that systolic blood pressure in SHR rats was markedly increased (Figure 1B), and hypertension (≥180 mmHg) was induced in SHR rats from 12 weeks of age. SHR rats with systolic blood pressure of approximately 200 mmHg and age-matched WKY rats (12 weeks of age) were used as the control for MCAo surgery. It has been reported that hypertensive SHR rats show increased infarct volume following MCAo compared with control WKY rats [5,36]. To determine the effects of hypertension on cerebral infarction due to MCAo, WKY and SHR rats with MCAo surgery were sacrificed 24 h post-injury. SHR rats had greater cerebral infarct volume in almost all brain slices versus WKY rats (Figure 2A,B). These results demonstrate that in SHR rats, MCAo surgery resulted in greater cerebral infarction volume compared with control WKY rats. It appears that hypertension is a risk factor modulating lesion volume after focal cerebral ischemia. Thus, lowering blood pressure may be neuroprotective in cerebral ischemic injury.

MCAo surgery has been widely used to investigate the pathogenesis and process of repair from focal cerebral ischemia, and it induces neurological deficiencies and motor dysfunction [5,21,22,23,35]. Barone *et al.* (1992) reported that forelimb flexion, muscle weakness, sensory motor deficits, decreased normal placement response, and decreased rota-rod and balance beam performance occurred following MCAo, and these were greater in SHR than in SD or WKY rats [36]. We also found typical neurological deficiencies, including flexion of the paretic forelimb, spontaneous circling, and decreased grip power of the paretic forelimb in the MCAo group in both WKY and SHR rats (Figure 3). The mNSS score was increased following MCAo surgery and decreased after forced exercise in the MCAo + Ex groups of WKY and SHR rats. Additionally, WKY rats showed larger improvements in mNSS scores than did SHR rats after forced exercise (Table 1). MCAo rats showed disruption of balancing ability, though it recovered after forced exercise in both WKY and SHR rats (Table 1). We confirmed motor dysfunction in both MCAo groups based on changes in hindlimb stride length, but it was significantly attenuated in both MCAo + Ex groups (Table 1). Thus, these results showed that hypertensive SHR rats had severe pathological changes and delayed recovery from neurological deficiency following focal cerebral ischemia and that forced exercise improved neurological deficiencies and motor dysfunction due to MCAo. Although there were no significant differences between WKY and SHR for beam-walking score and hindlimb stride width, it should be noted that more sensitive behavioral tests such as a reaching task might better demonstrate functional deficits between WKY and SHR rats.Caveolins are involved in the process of pathogenesis and repair after cerebral ischemia via interaction with other signaling molecules [23,24,25,35]. In particular, caveolin-1 has been implicated as a strong regulatory candidate in the pathogenesis of cerebral ischemic injury via modulation of NOS isoforms [22,23,24,25,35]. We found that caveolin-1, -2, and -3 mRNA levels were decreased in the MCAo group in WKY and SHR rats, but iNOS mRNA level of MCAo group was increased. These results indicate that caveolins and iNOS may be important causative factors in brain impairment at the molecular level after focal cerebral ischemia. It has been reported that caveolin-1 deficient mice showed a more severe cerebral infarction than did wild-type mice [35] and that MCAo-reperfusion led to an increase in iNOS expression and NO production as well as a decrease in caveolin-1 protein at the core and penumbra of the ischemic brain [23]. On the other hand, caveolin-1, -2, and -3 mRNA levels were increased by forced exercise, but iNOS mRNA levels decreased. No significant changes were noted in nNOS levels between any of the experimental groups. Endurance exercise results in changes in neuromuscular activities, energy metabolism, and hormonal responses, and the recruitment of different motor units, so it has been used in treating various pathological conditions affecting neurological and motor function [37]. BDNF expression was up-regulated in the hippocampus of rats after a swimming exercise [38]. Also, the Trk and PI-3 kinase/Akt pathways are activated by exercise and likely contribute to enhanced neuronal cell survival after exercise [39]. Additionally, phosphorylation of Trk receptors activates the Ras/MAPK pathway, which is related to the process of repair after cerebral ischemic injury [40]. These results suggest that forced exercise is beneficial in recovery from cerebral ischemia. Nevertheless, we confirmed that iNOS mRNA levels were significantly increased in the MCAo group of hypertensive SHR rats compared with WKY rats. Moreover, caveolin-1 and -2 mRNA levels were lowered, and iNOS mRNA levels increased in SHR and WKY rats after forced exercise. From these results, we concluded that hypertension may delay the process of repair after focal cerebral ischemic damage.

We found a significant decrease in caveolin-1 protein levels and an increase in iNOS and GFAP protein levels after MCAo in both WKY and SHR rats. The role of caveolin-1 in the CNS [41] in the context of neuronal plasticity [18] and Alzheimer's disease [17,42] is well known, and neurological abnormalities have been reported in caveolin-1 knock-out mice [43]. Additionally, caveolin-1 has a pivotal role in cerebral ischemic injury, blocking production of NO via iNOS [23,35]. NO aggravates neuronal damage after spinal cord injury, and NO produced by iNOS accelerates secondary damage to spinal tissue [44]. Thus, increased iNOS protein levels in the MCAo groups showed the presence of a secondary inflammatory response following focal cerebral ischemia. GFAP is the major intermediate filament of mature astrocytes, and its relatively specific expression in these cells suggests an important function; specifically, up-regulation of GFAP in the MCAo group indicates the presence of activated astrocytes in the ischemic brain [45]. In the present study, the caveolin-1 protein levels were up-regulated after forced exercise, while iNOS and GFAP protein levels were down-regulated in WKY and SHR rats. Although forced exercise attenuated these changes, SHR rats showed lower caveolin-1 protein levels and higher iNOS production than did WKY rats. Endothelial dysfunction has been described in SHR rats as well [2,4]. In support of this, the NO level was elevated in these rats compared to WKY rats [5,36]. Thus, modulation of iNOS via caveolin-1 might prevent secondary inflammation following cerebral ischemic injury. To investigate the activation of autophagy following focal cerebral ischemic damage, we assessed LC3-II and beclin-1 protein levels in WKY and SHR rats. There was no significant change in WKY rats, whereas the MCAo group of SHR rats showed elevation of LC3-II protein levels which was attenuated by forced exercise in these animals. MCAo rats showed elevation of beclin-1 protein levels, which were reduced after forced exercise in WKY and SHR rats. These results indicate that MCAo stimulated autophagic cell death, and these effects were partially reversed after forced exercise. Activation of autophagy occurs after focal cerebral ischemia and is associated with up-regulation of beclin-1 [30]. The autophagic process begins with the entrapment of material in a double-membrane vesicle named the autophagosome [28,29]. LC3 has been shown to be incorporated specifically into autophagic vesicles under conditions that induce autophagy, and thus serves as a marker for autophagosomes in mammalian cells [30,31]. There are two forms of LC3, LC3-I and LC3-II. LC3-I is cytosolic, whereas LC3-II is membrane bound, and the amount of LC3-II is correlated with the extent of autophagosome formation [31,32]. It has been reported that autophagy was activated in cardiac tissue in caveolin-1 deficient mice [33], indicating autophagy related to caveolin-1 expression. Class III PI3K-related autophagosome formation also involved a caveolae-associated binding protein in mammalian cells [34]. We confirmed activation of autophagy and down-regulation of caveolin-1 protein levels during focal cerebral ischemia. It remains unknown whether autophagy is advantageous or detrimental in the pathophysiology of cerebral ischemia. However, autophagy follows a physiological state or survival strategy by self-downsizing; eventually, autophagy leads to cell death. Thus, we suggest that optimizing forced exercise could help suppress focal cerebral ischemia-induced autophagy via enhancing caveolin expression, and this may represent a potential therapeutic strategy for cerebral ischemia.

## 4. Experimental Section

### 4.1. Experimental Animals

All animal experiments were performed according to a protocol approved by the guidelines of the Ethics Committee of Animal Experiments at Inje University. Every effort was made to minimize the number and suffering of animals used in our experiments. To confirm hypertension, 12 male WKY and SHR rats (180–210 g, 6 weeks of age) were purchased from Charles River (Japan). Body weight and blood pressure were monitored over 10 weeks. To induce focal cerebral ischemia, 24 male WKY and SHR (260–280 g, 12 weeks of age) were used. All rats were single-housed in opaque plastic cages (50.8 × 25.4 × 25.4 cm) at a controlled temperature (25 ± 1 °C), relative humidity (55% ± 10%), and light-dark conditions (12 h light/dark) before experimentation. Food and water were available *ad libitum*. 

### 4.2. Experimental Groups

**Figure 6:**
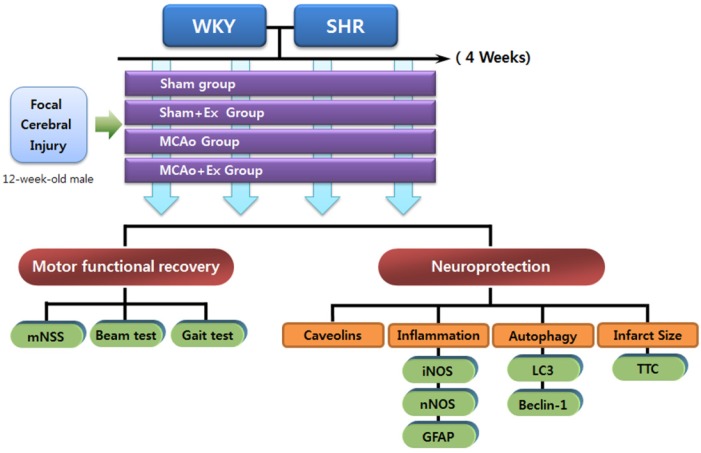
Schematic diagram of experimental design. WKY and SHR rats were randomly divided into four groups: Sham, Sham + Ex, MCAo, and MCAo + Ex groups. The brain tissue of the core infarction region was analyzed for expression of caveolins, inflammatory activation markers (NOS isoforms (iNOS and nNOS) and GFAP), and mediators of autophagic cell death (LC3 and beclin-1). Motor dysfunction due to MCAo was also evaluated.

As shown at Figure 1A, both WKY and SHR rats were randomly divided into four groups: Sham, sham with exercise (Sham + Ex), MCAo, and exercise after MCAo (MCAo + Ex). In the Sham group (*n* = 6), as a normal state without injury, exercise was not administered. The Sham + Ex group (*n* = 6) performed motor-driven treadmill exercise. In the MCAo group (*n* = 6), MCAo was induced, and exercise was not administered. In the MCAo + Ex group (*n* = 6), MCAo was induced, and the animals exercised on a motor-driven treadmill. Rats were sacrificed 28 days after injury, and brain tissue was obtained for histological and biochemical analysis (see Figure 6). 

### 4.3. Measurement of Blood Pressure

Blood pressure was measured using noninvasive tail cuffs and a programmed electrosphygmomanometer following the manufacturer’s recommendations (PowerLab/400, AD Instrument, Bella Vista, New South Wales, Australia). Two or three hours prior to taking blood pressure measurements, we handled each rat gently, and room temperature was maintained at 25–26 °C. For each rat, baseline blood pressure measurement was performed in six to seven consecutive measurements each day for 2–3 days. Blood pressure measurements were continuously monitored over an hour. 

### 4.4. Induction of Focal Cerebral Ischemia: MCAo

Focal cerebral ischemia was induced by the intraluminal suture MCAo method as previously described [20,21,22,23]. During the surgical procedure, body temperature was monitored continuously with a rectal probe and maintained at 36.5–37.0 °C with a thermostatically controlled water flow system. Animals were anesthetized with ketamine (50 mg/kg; Yunhan Corporation, Seoul, Korea) and xylazine (10 mg/kg; Rompun, Bayer Healthcare, Seoul, Korea), i.p. Under a surgical microscope (Olympus SZ-61TR, Tokyo, Japan), the right common (CCA), internal (ICA), and external (ECA) carotid arteries were exposed through a midline neck incision. The occipital artery branches of the ECA were then isolated and cauterized by electrical cautery (Bovie, Jungdo P & P, Korea). The CCA was occluded with a provisional ligation, and the ICA was isolated and carefully separated from the adjacent vagus nerve. Then, a microvascular clamp was placed on the ICA, and the ECA was dissected farther distally after ligation. A probe (*i.e.*, a 5-cm length of 4–0 monofilament nylon suture; AILEE Co., Busan, Korea), its tip rounded by heating near a flame and coated with silicone, was inserted via the ECA. The microvascular clamp was removed after confirming that the probe was directed toward the ICA. The probe was advanced into the ICA from 18 to 20 mm beyond the carotid bifurcation. Mild resistance indicated that the probe was properly lodged in the anterior cerebral artery and thus blocked blood flow to the middle cerebral artery (MCA). The 6–0 silk suture around the ECA stump was tightened around the intraluminal nylon suture to prevent bleeding, and muscle and skin were sutured. Reperfusion was allowed by pulling the probe back by 1 cm after 1 h. 

### 4.5. Protocol for Forced Treadmill Exercise

Starting three days after the operation, all experimental rats (Sham + Ex and MCAo + Ex groups) were trained to walk quadrupedally on the treadmill for 4 weeks twice a day (17:00 h and 22:00 h) for 30 min. Treadmill training consisted of quadrupedal treadmill stepping. Initially, rats were placed on the treadmill and the speed was started to their capabilities (7–10.5 m/min) during first 5–10 min for adaptation. If rats were adapted to walk quadrupedally on the treadmill, then the speed was increased to 20–25 m/min gradually to their capabilities after training. Rats typically started stepping when they experienced some small load on their hindlimbs. This training was performed 6 days a week for 4 weeks [44,46].

### 4.6. Modified Neurological Severity Score (mNSS)

Neurological deficiency was evaluated by a modified neurological severity score (mNSS), which shows neurological deficiencies graded on a scale of 0 to 18 (normal score: 0, maximal deficit score: 18). The mNSS is a composite of motor, sensory, reflex, and balance tests. In the injury severity scores, one point is awarded for the inability to perform the test or for the lack of a tested reflex; thus, a higher score is representative to a more severe injury [36]. Investigators were blind to the experimental groups during testing and analysis.

### 4.7. Beam-Walking Test

The beam-walking test was used to assess deficits in coordination and integration of motor movement, especially in the hindlimbs. The beam-walking apparatus consisted of a square beam (2.5 cm wide, 122 cm long, at a height of 42 cm). Performance was rated as follows: the rat was not able to stay on the beam, 0 points; the rat did not move, but was able to stay on the beam, 1 point; the rat tried to traverse the beam, but fell, 2 points; the rat traversed the beam with more than 50% footslips of the affected hindlimb, 3 points; the rat traversed the beam with more than one footslip, but less than 50%, 4 points; the rat had only one slip of the hindlimb, 5 points; the rat traversed the beam without any slips of the hindlimb, 6 points [22]. Investigators were blind to the experimental group assignment during testing and analysis.

### 4.8. Gait Analysis: Hindlimb Stride Width

The hind paws of tested animals were painted with non-toxic finger paint of different colors. The rats were then placed in a cardboard tunnel (10 × 10 × 85 cm) lined with a white strip of paper. The hindlimb stride width was defined as the distance between successive paw prints of different colors [43]. Investigators were blind to the experimental groups of the rats during testing and analysis.

### 4.9. Two Percent 2,3,5-Triphenyltetrazolium Chloride (TTC) Staining

The most frequently used tool for measuring the efficacy of putative neuroprotective compounds is 2,3,5-triphenyltetrazolium chloride (TTC) staining. The colorless TTC is enzymatically reduced to a red formazan product by dehydrogenases, which are abundant in mitochondria [21]. The 2% TTC (Sigma-Aldrich, St. Louis, MO, USA) solution was prepared in 37 °C phosphate buffer (0.2 M Na_2_HPO_4_ and 0.2 M NaH_2_PO_4_, pH 7.4–7.6) immediately before use. Rats were sacrificed, and the brains were removed and cut into 2-mm-thick coronal slices between the bregma levels of +4 mm (anterior) and −6 mm (posterior) using a rodent brain matrix (RBM-4000C, ASI instruments, Warren, MI, USA) [23]. The brain slices were incubated for 30 min in 2% TTC solution at 37 °C and tissue infarction appeared as a white area.

### 4.10. Isolation of Brain Tissue

All rats were anesthetized with ketamine (50 mg/kg) and xylazine (10 mg/kg), brains were removed 28 days after surgery and cut into coronal slices of 2-mm-thickness between the bregma levels of +4 mm (anterior) and −6 mm (posterior) using a rodent brain matrix (ASI instruments Inc., Warren, USA) [23]. Infarction core area appears as the white area like in the Figure 2, and we collected the tissue of infarct core area from all brain slices and used for analysis of gene and protein expression.

### 4.11. RNA Isolation and Reverse Transcription-Polymerase Chain Reaction (PCR) Analysis

Brain tissue was homogenized with 1 mL of TRIzol (Invitrogen, Carlsbad, CA, USA) to prepare total RNA. The RNA was reverse transcribed with oligo(dT)12–18 primers and M-MuLV reverse transcriptase (Invitrogen, Carlsbad, CA, USA); this reaction mix served as a template for PCR. To identify caveolin-1,-2, and -3, iNOS, nNOS, and GAPDH transcription, a PCR reaction mixture (50 μL) was made up consisting of 2.0 μL of cDNA synthesis mixture, 40 nM dNTPs, 10 pmol of sense and antisense primers, and 1.25 U of Taq polymerase (Takara, Tokyo, Japan) as previously described [44]. PCR was performed with denaturation at 95 °C for 1 min, annealing at 55 °C (caveolin-1) or 60 °C (caveolin-2, -3, iNOS, nNOS, and GAPDH) for 1 min, and extension at 72 °C for 1 min in each cycle, followed by a final 5 min extension at 72 °C with 40 cycles using a using a Px2 Thermal cycler (Thermo Electron Co., Waltham, MA, USA). Primer sequences are shown in Table 2. The expression densities of amplified bands were quantified with ImageJ v. 1.6 (NIH, Bethesda, MD, USA).

**Table 2:** Oligonucleotide primers used for RT-PCR in this study.

Gene	Primer Sequence(5′–3′)	Tm (°C)	Product Length (bp)	GenBank Accession No.
*Caveolin-1*	F: GATCAAGCTTATGTCTGGGGGCAAATAC	55	537	AY439333
	R: GATCGAATTCTCATATCTCTTTCTGC			
*Caveolin-2*	F: GATCAAGCTTATGGGGCTGGAGACCGAG	60	489	NM_016900
	R: GATCGAATTCTCAGTCGTGGCTCAGTTG			
*Caveolin-3*	F: GATCAAGCTTATGATGACCGAAGAGCAC	60	456	NM_007617
	R: GATCGAATTCTTAGCCTTCCCTTCGCAG			
*iNOS*	F: GTGTTCCACCAGGAGATGTTG	60	272	U03699
	R: GAAGGCGTAGCTGAACAAGG			
*nNOS*	F: TGGCAACAGCGACAATTTGA	60	71	NM_052799
	R: CACCCGAAGACCAGAACCAT			
*GAPDH*	F: GTATGACTCCACTCACGGCAAA	60	100	BC094037
	R: GGTCTCGCTCCTGGAAGATG			

### 4.12. Western Blot Analysis

Injured brain regions were homogenized in ice-cold lysis buffer, and protein concentrations were estimated by the Bradford assay (BioRad Laboratories, Richmond, CA, USA) as in a previous study [47]. Lysates were resuspended in Laemmli sample buffer and denatured at 95 °C for 10 min. Proteins were loaded (30 μg) onto a 15% polyacrylamide minigel and transferred to poly-vinylidene difluoride (PVDF) membranes (Millipore, Bedford, MA, USA). For immunoblotting, membranes were blocked with 5% non-fat dry milk in Tris-buffered saline (TBS) overnight at 4 °C and then incubated with primary antibodies (anti-caveolin and anti-GAPDH, 1:1000, Santa Cruz, Santa Cruz, CA, USA; anti-LC3, anti-beclin 1, anti-iNOS, and anti-GFAP, 1:1000, Cell Signaling, Beverly, MA, USA) overnight at 4 °C in 1× TBS, 5% w/v non-fat dried milk, and 0.1% Tween-20 (TBST). The membranes were washed three times for 5 min each in TBST and incubated with peroxidase-conjugated bovine anti-rabbit or anti-mouse IgG secondary antibody (1:5000; Santa Cruz, Santa Cruz, CA, USA). Proteins were visualized by enhanced chemiluminescence (ECL).

### 4.13. Statistical Analysis

All analyses were performed using the SPSS statistical package software, v. 18.0. Analysis of non-parametric measures was performed by the Mann-Whitney two tailed test for behavioral data. These values were represented by median value and range. All gene and protein expression differences were determined using one-way ANOVA and Tukey’s post hoc comparisons. Differences were deemed to be statistically significant when the *p*-value was < 0.05.

## 5. Conclusions

The central findings of this study are that hypertensive SHR rats with MCAo showed larger cerebral infarction size, critical neurological changes, and motor dysfunction compared with normotensive WKY rats. They also showed greater down-regulation of caveolin isoforms and up-regulation of iNOS and GFAP than did WKY rats. Additionally, forced exercise attenuated these changes and inhibited autophagic cell death after focal cerebral ischemic injury in WKY and SHR rats. Taken together, our findings provide evidence that improved motor and behavioral outcomes induced by forced exercise in MCAo-operated rats is associated with altered expression of caveolins and their interaction with inflammatory and autophagic signaling in ischemic brain regions.
